# The Impact of a 3-Month Behavioral Weight Loss Intervention on Psychological Flexibility and Depression

**DOI:** 10.3390/bs15060788

**Published:** 2025-06-07

**Authors:** Samantha J. Schram, Jason Lillis

**Affiliations:** 1 Department of Psychological Sciences, University of Connecticut, 406 Babbidge Rd, Unit 1020, Storrs, CT 06269, USA; samantha.schram@uconn.edu; 2 Weight Control and Diabetes Research Center, 196 Richmond St, Providence, RI 02903, USA

**Keywords:** psychological flexibility, behavioral weight loss, depression

## Abstract

A significant portion of individuals with overweight or obesity seek behavioral weight loss, which aims to modify behaviors and habits related to diet, physical activity, and lifestyle and has been shown to result in clinically meaningful weight loss and improved cardiometabolic health. While the physical outcomes of behavioral weight loss are well documented, relevant psychological processes and mental health outcomes are less studied. This secondary analysis examined psychological flexibility in relation to depression change and weight change in adults (N = 508) with a body mass index greater than 25 enrolled in a 3-month automated behavioral weight loss intervention. Psychological flexibility was assessed using the Comprehensive Assessment of Acceptance and Commitment Therapy Processes (CompACT), a tool based on Acceptance and Commitment Therapy (ACT) principles. The Patient-Reported Outcomes Measurement Information System (PROMIS) initiative Depression-Short Form, which collects information directly reported by patients, was used to measure levels of depression. Change in psychological flexibility was found to significantly moderate the positive relationship between weight change and change in depression over the course of a 3-month behavioral weight loss intervention, such that individuals with greater increases in psychological flexibility experienced greater reductions in depression levels when also losing weight. These results suggest that psychological flexibility may enhance the psychological benefits of behavioral weight loss and could be a useful target in a modified version of behavioral weight loss intervention.

## 1. Introduction

Obesity is a major public health concern impacting between 30.4% and 39.8% of adults in the United States (Baskin et al., 2005; Flegal et al., 2010; Hales et al., 2017; Ogden et al., 2020). Obesity has been linked to several significant physical health concerns (Williams et al., 2015; “Clinical Guidelines for Overweight and Obesity in Adults”, 1998), including cardiovascular disease, type 2 diabetes mellitus, hypertension, stroke, cancer, sleep apnea, liver and gallbladder disease, osteoarthritis (Guh et al., 2009; Nestle & Jacobson, 2000), poorer overall quality of life (Kolotkin et al., 2001; Stephenson et al., 2021), and adverse mental health outcomes (Sarwer & Polonsky, 2016; Clark et al., 2003; Cifuentes et al., 2022), including depression (Carpenter et al., 2000; Faith et al., 2002; Roberts et al., 2003; Stunkard et al., 2003).

More than one-half of adults with overweight or obesity living in the United States attempt to lose weight (Martin et al., 2018) for both health and appearance-related reasons (Levy & Heaton, 1993). Behavioral weight loss programs are widely implemented and structured evidence-based interventions that target changes in diet (i.e., caloric restriction), physical activity (i.e., increases in moderate-intensity activities), and self-monitoring behaviors (i.e., daily recording of weight, diet, and activity) (Olson et al., 2017). Behavioral weight loss programs have been shown to result in gradual and clinically meaningful weight loss typically over 3–12 months (Wadden & Butryn, 2003) and improved cardiometabolic health (Look AHEAD Research Group et al., 2013; Diabetes Prevention Program Research Group, 2002).

Of note, many individuals with overweight or obesity who engage in behavioral weight loss treatment experience depressive symptoms (Pagoto et al., 2007). While behavioral weight loss programs primarily target physical health outcomes, their psychological consequences, including changes in depression levels, remain inadequately understood; findings regarding the impact of behavioral weight loss on mental health outcomes are mixed; however, results are more consistent for depression (Jones et al., 2021). Although some literature has suggested that depressive symptoms are reduced due to weight loss (Dixon et al., 2003), most individuals who receive weight loss intervention remain with overweight or obesity or regain the weight they had lost (Loveman et al., 2011; Wing & Phelan, 2005), and thus remain targets of weight stigma and are prone to the negative mental health impact of living in a larger body. Given the mixed findings and complexities of the relationship between behavioral weight loss and mental health outcomes, there is a need for research that examines how participation in these interventions may influence depression levels, as well as the underlying mechanisms that support changes. A better understanding of this relationship would likely inform the development of more supportive, effective, and inclusive interventions that account for mental health outcomes alongside physical health improvements.

The psychological flexibility model (Levin et al., 2012) may provide a framework for understanding the potential psychological changes that occur during behavioral weight loss intervention. Psychological flexibility is the ability to act consistently with one’s stated personal values even when doing so would be accompanied by psychological distress (i.e., negative thoughts, emotions, or physiological experiences) (Hayes et al., 1999; Kashdan & Rottenberg, 2010). Psychological flexibility is associated with a broad range of mental (Gilbert et al., 2019; Lucas & Moore, 2020) and physical health (Gentili et al., 2019) clinical presentations and has been linked specifically to disordered eating (Masuda & Tully, 2012; Rawal et al., 2010), depression (Leahy et al., 2012; Masuda & Tully, 2012; Wang et al., 2023), and weight management (Lillis & Hayes, 2008).

Within the context of behavioral weight loss, individuals are tasked with making sustained changes related to their eating, physical activity, and other lifestyle behaviors (Olson et al., 2017). These changes can be difficult to maintain over time, and individuals with greater psychological flexibility may be more successful in adhering to the behaviors necessary for achieving their weight loss goals. On the other hand, engagement in behavioral weight loss interventions may also foster positive changes to psychological flexibility given the consistent practice of value-driven behavior change despite associated challenges and discomfort. Despite its potential relevance, relatively little research has examined the impact of behavioral weight loss treatment on psychological flexibility. However, related studies have found that higher levels of physical activity (Shen et al., 2024) and lower binge eating behaviors (Lillis et al., 2011; Finger et al., 2020), which are behaviors that are typically targeted by behavioral weight loss treatment, are related to psychological flexibility. Therefore, more research surrounding positive changes in psychological flexibility in the context of behavioral weight loss could help to improve intervention outcomes.

Additionally, a study of 468 treatment-seeking adults with overweight or obesity found that psychological flexibility may have served as a protective factor against binge eating for individuals with depressive symptoms (Godfrey et al., 2019). Bluett et al. (2016) found that pre-treatment levels of psychological flexibility longitudinally predicted improved quality of life and reduced eating disorder risk for 113 adolescent and adult female residential patients. Increases in psychological flexibility related to eating and physical activity have also been shown to partially mediate weight loss for 283 adults randomized to a 12-month acceptance-based or non-acceptance-based weight loss treatment (Schumacher et al., 2019). Together, these findings suggest the potential of psychological flexibility to have a positive impact on psychological functioning in the context of eating and weight-related concerns. Therefore, a clearer understanding of the impact of psychological flexibility on the relationship between weight loss and psychological well-being would likely help clarify the mechanisms through which behavioral interventions influence mental health outcomes and inform the development of more effective weight loss programs.

Given that behavioral weight loss requires a substantial amount of behavior change, including goal setting, intensive self-monitoring, meal planning and preparation, and increased physical activity, and is often linked to areas of great importance (i.e., health, well-being, the ability to provide for others; O’Brien et al., 2007; Mroz et al., 2018), it is possible that increases in psychological flexibility occur as a result of participating in behavioral weight loss intervention. Conversely, higher psychological flexibility may confer an advantage in terms of the ability to initiate and sustain the kind of large-scale behavioral changes required for successful participation in behavioral weight loss.

The current study is a secondary analysis of data from a 3-month automated online behavioral weight loss intervention designed to promote changes in eating, physical activity, and self-monitoring behaviors. This analysis aimed to explore the relationships between psychological flexibility, change in depression levels, and weight loss outcomes. The first aim was to examine whether baseline psychological flexibility predicted changes in BMI and depression following the invention. The second aim was to evaluate whether changes in psychological flexibility were associated with concurrent changes in BMI and depression. It was hypothesized that psychological flexibility (both at baseline and 3-month change) would be associated with changes in BMI and depression and would moderate the relationship between BMI change and change in depression.

## 2. Materials and Methods

### 2.1. Design

The current study was part of a larger randomized clinical trial (Lillis et al., 2021) in which all participants were enrolled in a 3-month online weight loss program before being randomly assigned to one of two maintenance interventions. The data used in the present analysis were drawn from the initial, single-arm phase of the trial. All procedures were reviewed and approved by the Lifespan Institutional Review Board (IRB), and written informed consent was obtained from all participants.

### 2.2. Participants

A total of 508 participants enrolled in the study and participated in the 12-week online weight loss program. The sample was 86.2% female and 84.6% white, with an average age of 54.6 ± 10.7 years. In order to be eligible for the study, participants had to be 25–70 years old and have a baseline body mass index (BMI) between 27.5 and 45 kg/m^2^, fluency in English at least sixth-grade reading level proficiency, and the ability to walk at least two city blocks without breaks. Participants were excluded if they were currently participating in another weight loss program, using weight loss medications, had lost ≥ 5% of their body weight in the past 6 months, were pregnant or planned to become pregnant, or had any medical or psychiatric condition that could interfere with safe participation or adherence to study procedures.

Given that this study is a secondary analysis, there was no a priori power analysis for the results reported in this manuscript. The larger randomized trial that includes these data was planned to have adequate power at N = 480 participants during the phase of the study analyzed in this manuscript, and therefore, the target N was exceeded.

### 2.3. Recruitment and Screening

Participants self-referred to the study via online or direct-mailed advertisements (i.e., not referred by a clinician). They completed a phone screen that determined BMI based on self-reported height and weight and provided health status information that was reviewed to assess eligibility. Eligible participants attended an orientation session via remote conferencing software (Zoom), where they received a detailed explanation of the study and provided informed written electronic consent.

### 2.4. Intervention

Participants took part in Rx Weight Loss (Thomas et al., 2015), a 12-week online behavioral weight loss intervention based on the Diabetes Prevention Program (The Diabetes Prevention Program (DPP) Research Group, 2002). The program included 12 10–15 min weekly expert-led interactive multimedia sessions on topics such as energy balance, exercise goals, problem-solving strategies, and navigating food choices when dining out. Rx Weight Loss has shown strong effectiveness across many studies, consistently producing safe and clinically significant weight losses of approximately 5% over 12 weeks (Thomas et al., 2015).

Based on their initial body weight, participants were assigned individualized daily calorie goals ranging from 1200 to 1800 kcal per day (with 25% of caloric intake from fat), aiming for a weekly weight loss of 1–2 pounds. They were also encouraged to progressively increase their physical activity to 200 min per week by the end of the program.

As part of the intervention, participants were instructed to log their calorie intake, exercise duration, and weight on the study website daily and received computer-generated feedback at the end of each week to support adherence to their dietary, weight, and activity goals. The mean number of weeks participants self-monitored was 8.7 ± 4.0.

### 2.5. Measures

All measures were collected at baseline and then at 3 months (post-treatment). Data collection spanned from 2020 to 2022. Participants completed questionnaires remotely through online surveys, and study staff collected anthropometric data in person.

#### 2.5.1. Anthropometric

BMI (kg/m^2^) was calculated using weight and height measurements obtained through standardized procedures. Weight was measured with a digital scale, measured to the nearest 0.1 kg, and height was measured to the nearest millimeter with a stadiometer.

#### 2.5.2. Psychological Flexibility

Psychological flexibility was measured using the Comprehensive Assessment of Acceptance and Commitment Therapy Processes (CompACT; Francis et al., 2016), a 23-item questionnaire that assesses psychological flexibility across three subscales: (1) Openness to Experience (OE; “One of my biggest goals is to be free from painful emotions”), (2) Behavioral Awareness (BA; “Even when doing the things that matter to me, I find myself doing them without paying attention”), and (3) Valued Action (VA; “I act in ways that are consistent with how I wish to live my life”). Responses are given using a Likert scale ranging from 0 (“strongly disagree”) to 6 (“strongly agree”). Twelve items are reverse scored prior to summing all response items to compute total and subscale scores. Total scores range from 0 to 138, with higher scores indicating greater psychological flexibility. The CompACT is valid for use as a full-scale single score (representing a general measure of psychological flexibility) and separately as the individual components described above. Given the nature of the current study and the fact that psychological flexibility was not a target of the intervention, the full-scale single score was used in analyses for this exploratory study. A more fine-grained examination of the components of psychological flexibility is more appropriate for interventions targeting psychological flexibility. The CompACT has demonstrated good internal consistency and has previously shown that higher levels of psychological inflexibility were associated with decreased health and well-being and increased distress (Francis et al., 2016). In the current sample, the total CompACT scale demonstrated good internal consistency (α = 0.853). Internal consistency was acceptable for the OE subscale (α = 0.766) and good for the BA (α = 0.802) and VA (α = 0.824) subscales.

#### 2.5.3. Depression

Depression was assessed using the National Institute of Health (NIH) Patient Reported Outcomes Measurement System (PROMIS) initiative Depression-Short Form (DeWalt et al., 2007). This measure includes four self-report items (e.g., “I felt worthless”) rated on a 5-point Likert scale ranging from 1 (“Never”) to 5 (“Always”). Total scores are calculated by summing item responses and range from 4 to 20, with higher scores reflecting higher depression levels. The PROMIS measure uses item response theory to enhance precision across a wide range of symptom severity. Given that the current study was part of a larger randomized clinical trial that includes many measures, the PROMIS measure was chosen for its brevity and low participant burden. In addition to its efficiency, this measure has demonstrated good reliability and validity in previous literature (DeWalt et al., 2007) and showed excellent internal consistency for the current sample (α = 0.901).

#### 2.5.4. Statistical Analysis

All analyses were performed using IBM SPSS Statistics software version 29 for PC in 2024. Baseline means and standard deviations were calculated for BMI, psychological flexibility, and depression for the full sample. Bivariate correlations were calculated between baseline levels and change in psychological flexibility as it relates to baseline levels and change in BMI and depression. Two multiple linear regressions were performed to examine the potential moderating effect of (1) baseline psychological flexibility and (2) change in psychological flexibility on the relationship between change in BMI and depression. In both analyses, pre-to-post change in BMI was an independent variable and change in depression was the dependent variable. In analysis one, baseline psychological flexibility was included as a covariate, and the interaction term of baseline psychological flexibility × BMI change was also included in the model. In analysis two, pre-to-post change in psychological flexibility was included as a covariate, and the interaction term of psychological flexibility change x BMI change was also included in the model.

## 3. Results

Participants had a mean baseline BMI of 34.74 (*SD* = 4.65). Table 1 shows the means and standard deviations for BMI, psychological flexibility scores, and depression scores at baseline and post-treatment.

Correlations between psychological flexibility scores and BMI and depression scores are shown in Table 2. Baseline psychological flexibility showed a medium, negative correlation with baseline depression scores (*r* = −0.545, *p* < 0.001) and a low, positive correlation with change in depression (*r* = 0.187, *p* < 0.001). Change in psychological flexibility showed a low, negative correlation with change in BMI (*r* = −0.200, *p* < 0.001) and change in depression scores (*r* = 0.244, *p* < 0.001). All other correlations were not statistically significant and negligible in magnitude.

Table 3 shows the multiple regression results. For analysis one, the overall model was significant (F = 9.24, *p* < 0.001, r^2^ = 0.08), with BMI change being an independent predictor of depression change such that reductions in BMI were associated with reductions in depression score. No effect was found for baseline psychological flexibility or the interaction term, suggesting that baseline psychological flexibility had no impact on depression change and also did not moderate the association between BMI change and change in depression. For analysis two, the overall model was significant (F = 15.27, *p* < 0.001, r^2^ = 0.13), and the only significant predictor was the interaction between BMI change and psychological flexibility change, suggesting that reduction in depression was dependent on the interaction of weight loss and increased psychological flexibility (i.e., a moderation effect).

## 4. Discussion

The present study examined the relationship between psychological flexibility and changes in BMI and depression symptoms in the context of a 3-month online behavioral weight loss intervention. The results were consistent with our hypotheses that baseline psychological flexibility and change in psychological flexibility would be significantly associated with changes in depression symptoms. These findings align with the growing literature that suggests that higher psychological flexibility and improvements in psychological flexibility over time may contribute to better psychological well-being and mental health outcomes in the general population (Bond & Bunce, 2000; McCracken et al., 2021). Consistent with our hypothesis, increases in psychological flexibility from baseline to the 3-month follow-up were associated with concurrent weight loss. Conversely, the results did not support our hypothesis that baseline psychological flexibility would be associated with greater weight loss. These findings suggest that behavioral changes enacted during behavioral weight loss treatment, such as self-regulation, goal setting, increased physical activity, or diet changes, may positively impact psychological flexibility processes, as opposed to the other way around.

Consistent with hypotheses, change in psychological flexibility moderated the association between weight loss and reduced depressive symptoms, such that reductions in depressive symptoms were more likely in individuals who both lost more weight and showed greater increases in psychological flexibility. When the interaction term was included in the model, weight change alone no longer significantly predicted change in depression score. Given the exploratory nature of this study, this result should be interpreted with caution. However, one possibility is that the psychological benefits of losing weight are not fully inherent to weight loss and may require changes in key psychological mechanisms to confer psychological benefits.

Given that behavioral weight loss requires a substantial amount of behavioral change to be successful, often changes that are hard to implement and sustain, it is not surprising that changes in psychological flexibility would be observed. Psychological flexibility, at its core, is doing what matters to oneself when doing so is psychologically challenging (i.e., in the presence of unwanted or negative thoughts, feelings, or bodily sensations). Despite not being directly targeted, behavioral weight loss intervention may naturally foster changes in psychological flexibility in some. Conversely, in this study, some individuals are losing weight and are not meaningfully increasing psychological flexibility, contributing to reduced psychological benefits with respect to weight loss. Again, caution is needed here, and this result is best seen as hypothesis generating for a study designed to test the potential of this moderating effect more robustly.

Future research could explore whether specific behavior changes lead to changes in psychological flexibility, as increased psychological flexibility has been shown to benefit individuals engaged in weight loss (Godfrey et al., 2019). Furthermore, psychological flexibility could be added as a specific target to a behavioral weight loss program to ascertain whether gains can be enhanced and thus also increase effect sizes for outcomes of interest, including weight loss and psychological well-being. Finally, given that behavior changes that accompany weight loss wane over time, and weight regain is common, it would be useful to know whether there is any durability to changes in psychological flexibility resulting from participating in a weight loss intervention.

The study strengths include using a validated weight loss intervention program, a large sample size, blind assessors, and standardized and objective weight measurement. This study was limited by a homogenous sample of mostly white middle-aged women. The intervention relied on self-reporting of weight, exercise duration, and daily caloric intake, and there were no ways in which the research team could verify if participants had followed the provided recommendations. Additionally, the use of self-reporting measures for depression symptoms and psychological flexibility could have led to self-report bias. Lastly, it should be noted that the current study occurred during the COVID-19 pandemic. The pandemic did not impact the delivery of the intervention since it was remotely delivered as designed. However, it is well understood that COVID-19 had a widespread impact on mental health, study procedures for some participants had to be modified during highly contagious periods (e.g., in-person assessments were reduced in scope or performed outside with limited contact), and it is unknown if and how that might have affected the outcomes of the study. While these effects were not directly assessed, they may limit the generalizability of findings and should be considered when interpreting results as depression symptoms increased in the general population during the pandemic (Ettman et al., 2020). Future research should examine the role of psychological flexibility in behavioral weight loss treatment, using a more diverse sample, a priori hypotheses, and a more robust assessment of psychological flexibility.

The present study highlighted the role of psychological flexibility in improving depression symptoms in the context of a 3-month online behavioral weight loss program. The findings highlight the importance of further research on the integration of interventions that target psychological flexibility into behavioral weight loss treatments, especially for individuals with comorbid depression and overweight or obesity. Future research is also necessary to explore how sustained changes in psychological flexibility influence long-term weight loss and mental health outcomes.

## Figures and Tables

**Table 1:** Baseline and post-treatment means and standard deviations.

	Full Sample (N = 508)
Baseline BMI	34.74
*SD*	4.65
Post-Treatment BMI	32.72
*SD*	4.78
Baseline Psychological Flexibility	91.30
*SD*	17.97
Post-Treatment Psychological Flexibility	95.76
*SD*	19.97
Baseline Depression	6.34
*SD*	3.08
Post-Treatment Depression	5.62
*SD*	2.58

**Table 2:** Correlations between psychological flexibility, BMI, and depression.

	Baseline BMI	BMI Change	Baseline Depression	Depression Change
Baseline Psychological Flexibility	−0.081	−0.054	−0.545 **	0.187 **
Psychological Flexibility Change	−0.016	−0.200 **	0.063	−0.244 **

** *p* < 0.001.

**Table 3:** Linear regression model results for 3-month change in depression.

Variable	Coefficient	Standard Error	*p*-Value
*Analysis 1*			
Intercept	−0.85	1.15	0.461
BMI change	1.09	0.49	0.027
Baseline psychological flexibility	0.01	0.01	0.429
BMI chg × BL PsyFlex	−0.01	0.01	0.103
*Analysis 2*			
Intercept	−0.04	0.21	0.856
BMI change	0.14	0.09	0.136
Psychological flexibility change	0.01	0.01	0.537
BMI chg x PsyFlex chg	0.02	0.01	<0.001

## Data Availability

The data presented in this study are available on request from the corresponding author due to ongoing data collection.
